# COVID-19 epidemic in the US—A gateway to screen for tuberculosis, HIV, viral hepatitides, Chagas disease, and other neglected tropical diseases among Hispanics

**DOI:** 10.1371/journal.pntd.0008953

**Published:** 2020-12-18

**Authors:** Jonathan Schultz, Peter Hyson, Daniel B. Chastain, Amal A. Gharamti, Carlos Franco-Paredes, Andrés F. Henao-Martínez

**Affiliations:** 1 Department of Medicine, Division of Infectious Diseases, University of Colorado Denver, School of Medicine, Aurora, Colorado, United States of America; 2 Department of Clinical and Administrative Pharmacy, University of Georgia College of Pharmacy, Albany, Georgia, United States of America; 3 Department of Internal Medicine, American University of Beirut, Beirut, Lebanon; 4 Hospital Infantil de México, Federico Gómez, México City, México; Sacro Cuore Hospital, ITALY

## Introduction

Hispanics are a very heterogeneous ethnic group with multiple backgrounds. The United States Census Bureau estimated that Hispanics reached 60.6 million in 2019. Most of the Hispanics are of Mexican origin, followed by Puerto Rican, Cubans, Salvadorans, and Dominicans. These numbers are underestimated, as there are migrants with a non-legal status and don’t appear in the census. They represent hidden populations, which can have important implications for the detection and control of transmissible diseases. Many of them have immigrated to the US escaping prosecution, political or civil unrest, and extreme poverty. Human immunodeficiency virus (HIV), tuberculosis (TB), and neglected tropical diseases (NTDs) have been a source and perpetuation of poverty in Latin America. Many of these infections are asymptomatic or latent and do not manifest clinically until many years later. As healthcare professionals currently treating and managing patients with Coronavirus Disease 2019 (COVID-19), we have realized (1) the disproportionate burden of disease among disadvantaged minority groups including Hispanics; and propose (2) a unique opportunity to link into care and screen patients for other infectious diseases of poverty including HIV, TB, and NTDs. This opportunity poses challenges for the US healthcare system, and we should perform cost-effective analyses and elaborate strategies to continue screening and linking people regardless of the current pandemic.

Compared with the general US population, Hispanics in the US have a greater proportion of foreign-born individuals (31% versus 13%), are younger (average age, 27 versus 37 years), hold fewer bachelor’s degrees (13% versus 28%), are less likely to be English proficient (65% versus 91%) or US citizens (74% versus 93%), have lower annual household income (US$40,000 versus US$49,800), have higher poverty rates (25% versus 15%), and are less likely to have health insurance (69% versus 84%) [1]. These demographic characteristics along with other determinants of health—living in densely populated areas, racial housing segregation, living in neighborhoods with limited access to medical facilities, greater use of public transportation, living with multifamily households, and overrepresentation in jails, prisons, shelters, and detention centers—makes Hispanics/Latinos more susceptible to acquire and develop worse outcomes with COVID-19 [2].

COVID-19 has disproportionally affected minority groups in the US including African Americans, Native Americans, and Hispanics. Centers for Disease Control and Prevention (CDC) racial and ethnic analyses have shown rates of COVID-19–associated hospitalizations—per 100,000 people—are 1.8, 1.7, and 1.4 -fold higher among Native Americans, African Americans/Blacks, and Hispanics, respectively, compared to non-Hispanic Caucasians, despite widespread underreporting [3]. Latin communities (counties with at least a quarter of Latin population) across the US have recorded an increase of 32% of COVID-19 cases compared to 15% in other communities [4]. Hispanics in Colorado, despite representing 21% of the population, account for 28% to 38% of the cases. We aim to briefly review the screening of common infectious diseases affecting Hispanics, immigrants, and other disadvantaged populations living in the US during the current COVID-19 pandemic.

## HIV/AIDS

**Fig 1:**
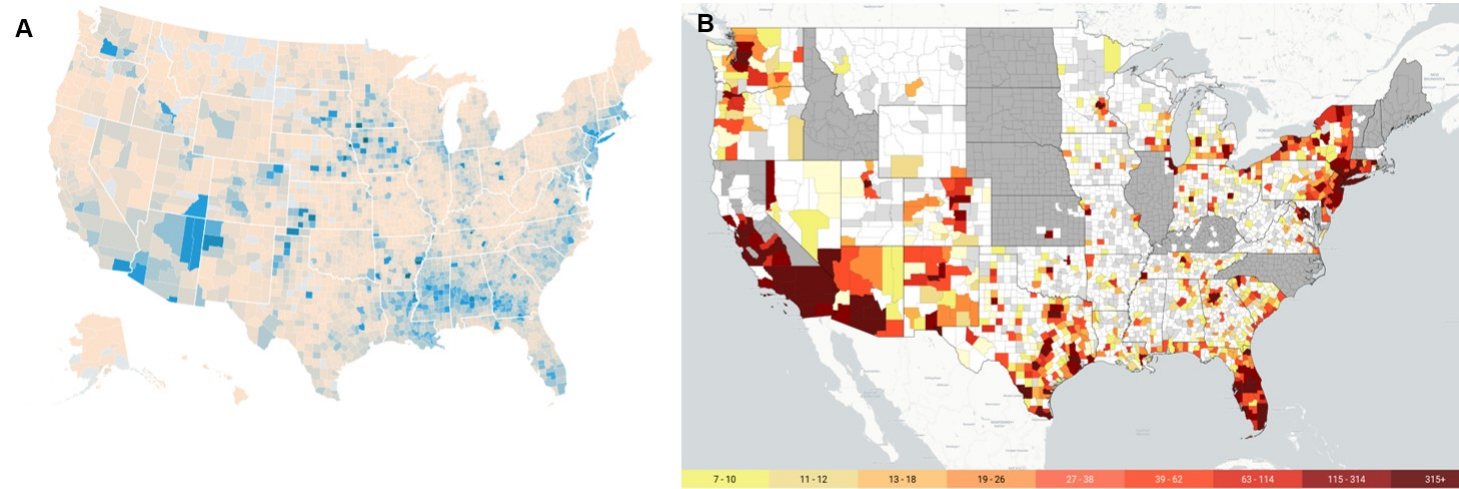
COVID-19 and HIV prevalence by US county. (A) Confirmed COVID-19 infections per 100K per county (source: USAFACTS.org). (B) Number of Hispanics/Latinx persons living with HIV, 2016 (source: AIDSVu.org). COVID-19, Coronavirus Disease July, 2019.

Unfortunately, like other infectious diseases linked to stigma, HIV has also concentrated in minority groups. By the end of 2016, approximately a quarter million of Hispanics had HIV [5]. In 2018, 37,968 people received an HIV diagnosis, of which 7,653 were Hispanics. According to the CDC, Hispanics accounted for 26% of new HIV infections in 2017, an increase from 20% in 2009, despite representing only 16.7% of the US population. More than 106,000 US Hispanics with AIDS have died since the beginning of the epidemic, including about 2,863 in 2016. The prevalence of HIV (per 100,000 population) for Hispanics/Latinos (575.7) is almost 3 times the rate for whites (198.1), and it increases to 4.5 times among Hispanic/Latinos women [5]. Hispanics are also more likely to present with advanced HIV infection requiring hospitalization and AIDS-defining illnesses [1]. Based on an age-adjusted percent distribution of adults in the US, up to 54.7% of Hispanics have never been tested for HIV [6]. During this pandemic, we have seen firsthand, Hispanics presenting with acute COVID-19 pneumonia to later be diagnosed with *Pneumocystis jirovecii* pneumonia coinfection and advanced AIDS. Hispanics suffering from COVID-19 also commonly share risk factors and social determinants for HIV (see overlap in Fig 1). Evaluating Hispanics and other minority groups with COVID-19 during their hospitalizations or any other healthcare setting represent an incredible opportunity for earlier diagnosis of HIV, linkage to care, and prompt initiation of antiretroviral therapy. The US Preventive Task Force (USPSTF) recommends clinicians to screen for HIV infection in all pregnant persons, adolescents, and adults aged 15 to 65 years of age. Fear of stigma, discrimination, and lack of access to healthcare are among the main barriers to screen for HIV among Hispanics. Strategies to enhance community HIV knowledge and awareness initiatives have been effective in overcoming HIV barriers to prevention among African migrants in Spain [7].

## Tuberculosis

TB continues to impact Hispanics/Latinos in a greater proportion compared to other ethnic groups. In 2018, TB disease was diagnosed among 2,617 Hispanics in the US, at a rate 8 times higher for Hispanic/Latinos compared to non-Hispanic whites [8]. Screening and treatment of latent tuberculosis infection (LTBI) remain a critical public health measure to decrease the burden of disease in vulnerable populations. The USPSTF recommends screening for LTBI in high-risk populations, such as persons living with HIV, diabetes mellitus, born in foreign countries with high TB incidence, and residence in shelters or correctional facilities, which frequently affect the Hispanic population [9,10]. Although there is not defined timing for LTBI screening in the US guidelines, the first years in the host country are usually the hardest, especially if they lack social support networks and have language barriers. Taking the opportunity of screening COVID-19 patients for other infections would allow establishing the first contact between these “naïve patients” and the healthcare system. The management of Hispanics diagnosed with COVID-19 creates a unique opportunity to identify those at increased risk of LTBI and provide recommended screening and linkage to care if positive. Ruling out active pulmonary TB infection remains essential in at-risk Hispanic patients presenting with atypical COVID-19 pneumonia.

## Chagas disease and other NTDs

Chagas disease is a parasitic infection caused by *Trypanosoma cruzi* and transmitted most commonly through the bite of its vector, the kissing bug (Triatomines). Chagas disease has infected around 6 million people in Latin America. Around 300,000 people or more live with Chagas disease in the US. Since the infection is only endemic in Latin America, Hispanic/Latin immigrants or descendants from infected Hispanic mothers are at risk of infection. Although epidemiological studies have documented autochthonous infection in the US, its relevance and impact are not well established. Untreated, Chagas infection leads to congenital infection in pregnant women and the development of cardiomyopathy at a rate of 2% annually in endemic countries [11]. Although we lack clear epidemiological, screening, and prevention cost-effectiveness guidelines of Chagas disease in the US, prevention and control of Chagas disease must be a priority. Treatment of Chagas disease decreases the risk of congenital infection and may slow the rate of cardiomyopathy development [12,13]. Furthermore, a decision-analytic model showed that universal screening of Chagas disease among pregnant women in the US would be cost-effective [14]. Finally, European models have shown that screening for Chagas disease among asymptomatic Latin Americans living in Europe is cost-effective [15,16]. We have also treated Hispanic COVID-19 patients, only later to discover Chagas disease contributing to their comorbidities and linked them to infectious diseases care. Disadvantaged and marginalized Hispanic populations surviving COVID-19 can widely benefit from screening, early diagnosis, and treatment of Chagas disease. Despite the absence of US guidelines, we recommend Chagas screening among asymptomatic and pregnant patients of Hispanic origin. We usually start with any commercial *T*. *cruzi* IgG test, followed by confirmatory serologic testing performed at CDC. Patients living with Chagas disease are subject to stigma. They also commonly have language barriers and a lack of healthcare access, which impose big challenges to any screening program. The use of multidimensional frameworks to analyze the barriers to diagnosis and treatment of Chagas disease can aid in organizing effective public health responses against the disease [17].

Although not exclusive of Hispanics/Latins, treating clinicians should evaluate the screening of additional NTDs such as schistosomiasis and strongyloidiasis or other soil-transmitted helminths (STHs) among other minority groups or immigrants in the presence of appropriate risk factors. The use of corticosteroids—a well-known risk factor for *Strongyloides* hyperinfection—is making strongyloidiasis screening and treatment crucial in the management of COVID-19 infection in patients at risk [18]. NTDs and COVID-19 disproportionately affect communities experiencing poverty and hospital admission. This is an excellent opportunity to screen for these underrecognized coinfections.

## Viral hepatitides

Hispanics are 60% more likely to die from viral hepatitides than whites. Despite an effective vaccine, hepatitis A virus (HAV) has increased by 140% from 2011 to 2017 based on CDC data, mostly among persons experiencing homelessness or those who use drugs and outbreaks among vulnerable populations. Homelessness rates among Hispanics double those from whites. Hispanic children in the US can be more susceptible to food-borne exposures during travel to Mexico[19]. The Advisory Committee on Immunization Practices recommends routine HAV vaccination of children, adolescents, and adults at risk for infection or severe disease.

Hepatitis B virus (HBV), also with an effective vaccine, is more common among persons born outside the US; however, it can be easily treated and managed [20]. Screening for HBV is recommended in high-risk populations, which are those more often presenting with COVID-19. These high-risk groups include persons born in countries with a high prevalence of HBV infection, HIV–positive patients, injection drug users, men who have sex with men, and household contacts or sexual partners of persons with HBV infection.

Hepatitis C virus (HCV) has also been increasing rapidly in the US, which can become a chronic infection that increases the risk of cirrhosis and hepatocellular carcinoma. Despite having lower rates of HCV, Hispanics were 40% more likely to die from that disease than non-Hispanic whites in 2015. HCV screening is recommended for all asymptomatic adults aged 18 to 79 years without known liver disease.

## Other noncommunicable diseases

Evaluation of Hispanics/Latins with COVID-19 in hospital settings has often uncovered the diagnosis of hypertension and new-onset diabetes mellitus. Both chronic conditions are known to modulate the susceptibility to infection and increase the risk for more severe COVID-19 and poor outcomes [21]. Managing any type of patients during COVID-19 should raise the opportunity to screen for chronic diseases and facilitate linkage to care. Hispanics carry a prevalence of diabetes mellitus type 2 of about 17%, compared to non-Hispanic whites (8%). Hispanic adults also reach a prevalence of 22% of hypertension.

It is important to recognize health inequalities and barriers that migrants and other minority groups have to face to access the US healthcare systems and, subsequently, to be screened and treated. Several experts have pointed out the efficacy of transversal and community-based interventions to deal with NTDs and other diseases [22,23]. It is crucial to adapt the interventions to each collective enhancing the role of primary healthcare settings to overcome those barriers. We also should not neglect an optimal medical follow-up of chronic diseases during the COVID-19 pandemic [24].

The use of preventive care measures during COVID-19 hospitalizations are underutilized. However, maximizing opportunities for preventative measures during the hospital stay or other healthcare settings represents a unique and moral obligation to screen for infections and chronic diseases that affect disadvantaged populations. Appropriate implementation of prevention strategies can lead to a decrease in future hospitalizations, increase cost savings, and improve clinical outcomes [25]. The current COVID-19 pandemic is an occasion to implement screening protocols, which are already recommended by guidelines in the US. To tackle communicable diseases with great health and economic repercussions—including COVID-19—the best tool we have at our disposal is to implement a universal healthcare system, in which access to care and testing is free.

The COVID-19 pandemic has unveiled chronic social inequalities and health disparities among minority groups. Although common preventive measures should be implemented for all patients, vulnerable groups like Hispanics/Latinos, immigrants, or those experiencing poverty who present to care with COVID-19 deserve appropriate infectious disease screening. We should continue efforts to screen for communicable diseases among disadvantaged minority groups including Hispanics, independent of whether infected or not with the Severe Acute Respiratory Syndrome Coronavirus 2 (SARS-CoV-2).
